# The Precarious State of the Liver After a Fontan Operation: Summary of a Multidisciplinary Symposium

**DOI:** 10.1007/s00246-012-0315-7

**Published:** 2012-04-26

**Authors:** Jack Rychik, Gruschen Veldtman, Elizabeth Rand, Pierre Russo, Jonathan J. Rome, Karen Krok, David J. Goldberg, Anne Marie Cahill, Rebecca G. Wells

**Affiliations:** 1 Division of Cardiology, Single Ventricle Survivorship Program, The Cardiac Center at the Children’s Hospital of Philadelphia, 34th Street and Civic Center Boulevard, Philadelphia, PA 19104 USA; 2 Department of Congenital Heart Disease, Southampton University Hospital, Southampton, UK; 3 Division of Gastroenterology, The Children’s Hospital of Philadelphia, Philadelphia, PA USA; 4 Department of Pathology, The Children’s Hospital of Philadelphia, Philadelphia, PA USA; 5 Division of Gastroenterology, Hospital of The University of Pennsylvania, Philadelphia, PA USA; 6 Division of Interventional Radiology, The Children’s Hospital of Philadelphia, Philadelphia, PA USA; 7 Department of Pediatrics, Perelman School of Medicine, University of Pennsylvania, Philadelphia, PA USA; 8 Department of Pathology, Perelman School of Medicine, University of Pennsylvania, Philadelphia, PA USA; 9 Department of Radiology, Perelman School of Medicine, University of Pennsylvania, Philadelphia, PA USA; 10 Department of Medicine, Perelman School of Medicine, University of Pennsylvania, Philadelphia, PA USA

**Keywords:** Decreased cardiac output, Elevated venous pressure, Fontan operation, Hepatic fibrosis, Liver

## Abstract

As the cohort of survivors with the single-ventricle type of congenital heart disease grows, it becomes increasingly evident that the state of chronically elevated venous pressure and decreased cardiac output inherent in the Fontan circulation provides the substrate for a progressive decline in functional status. One organ at great risk is the liver. Wedged between two capillary beds, with the pulmonary venous bed downstream, which typically has no pulsatile energy added in the absence of a functional right ventricle, and the splanchnic bed upstream, which may have compromised inflow due to inherent cardiac output restriction characteristic of the Fontan circulation, the liver exists in a precarious state. This review summarizes a consensus view achieved at a multidisciplinary symposium held at The Children’s Hospital of Philadelphia in June 2011. The discussion includes current knowledge concerning the hemodynamic foundations of liver problems, the diagnostic tools available, the unique histopathology of the liver after the Fontan operation, and proposed mechanisms for hepatic fibrosis at the cellular level. At the completion of the symposium, a consensus recommendation was made by the authors’ group to pursue a new prospective protocol for clinical evaluation of the liver for all patients in our practice 10 years after the Fontan operation.

As part of a systematic management strategy, the Fontan operation has allowed thousands of patients born with the single-ventricle type of congenital heart disease to survive. Advances in diagnostic capacities, surgical techniques, and perioperative care, as well as overall better understanding of the circulatory pathophysiology have contributed to major improvements in outcome. Survival through surgery and into the early years of life currently is expected for most individuals born with this condition [53]. As the number of survivors into adolescence and early adulthood continues to increase, questions have arisen concerning the capacity of the Fontan circulation to sustain a good quality and a normal duration of life.

The Fontan operation directs the systemic venous return into the pulmonary circulation, allowing for passive venous flow into the lungs without ventricular propulsion. This achieves the goal of reduced intracardiac mixing and improves systemic oxygen saturation, although with a number of physiologic limitations. After the Fontan operation, systemic venous pressure is obligatorily elevated. Limited preload filling of the systemic ventricle due to a missing pulmonary ventricle contributes to low stroke volume and diminished cardiac output [24]. Venous congestion and decreased perfusion (i.e., diminished oxygen delivery) are the hallmarks of this circulation.

As the cohort of survivors grows, it is increasingly evident that the state of chronically elevated venous pressure and decreased cardiac output provides the substrate for a progressive decline in functional status. Arrhythmia, valvular insufficiency, and ventricular dysfunction are common [4, 41]. Complications directly related to an impaired cardiovascular system such as reduced exercise capacity are noted [26]. In addition, there is growing evidence for a multitude of end-organ dysfunctions. Poor somatic growth and development, delayed pubertal maturation, increased risk of thromboembolism, peripheral venous insufficiency, protein-losing enteropathy, and plastic bronchitis are being identified with increasing frequency after the Fontan operation [10, 12, 45, 54, 55, 63].

One organ at great risk is the liver. In June 2011, a multidisciplinary symposium was held at The Children’s Hospital of Philadelphia to discuss the problem of the liver after the Fontan operation. The participants in this symposium included experts from both the pediatric and adult fields of congenital heart disease, cardiothoracic surgery, heart failure, and gastroenterology/hepatology, The group reviewed current knowledge concerning the hemodynamic foundations for liver problems, the diagnostic tools available, the unique histopathology of the liver after the Fontan operation, and proposed mechanisms for hepatic fibrosis at the cellular level. At the completion of the symposium, a consensus recommendation was made to pursue a new, prospective protocol for clinical evaluation of the liver. The following summary reviews our discussions concerning the precarious state of the liver after the Fontan operation.

## Impact of the Fontan State on Hepatic Circulatory Physiology

The Fontan patient, because pulmonary blood flow is passive, experiences an obligate elevation in systemic venous pressure compared with normal individuals. Similarly, because ventricular filling is limited by the passive flow of blood through the pulmonary circulation, the cardiac output in Fontan patients is diminished, and the ability to increase cardiac output in response to metabolic demands is substantially diminished [59]. These physiologic derangements become progressively more severe with each passing year [42].

In individuals with normal heart anatomy and function, hepatic blood flow accounts for approximately 25 % of the cardiac output. Approximately one-fourth of this flow is fully oxygenated arterial afferent supply via the hepatic artery. The remainder is deoxygenated blood at venous pressure (average, 6 mmHg) via the portal vein. Importantly, no capacity exists for autoregulation of portal venous flow. It is dependent on the mesenteric circulation and directly related to the gradient between portal and hepatic venous pressures.

The hepatic artery, which exhibits classic arterial autoregulation, is responsible for all autoregulation of hepatic blood flow. This autoregulation results in what has been termed the “hepatic arterial buffer response” (HABR). Decreased portal flow is buffered by increased hepatic arterial flow. It is estimated that the hepatic artery is capable of buffering a 30 % to 60 % decrease in portal inflow [14].

Very little data exist on hepatic circulation in the Fontan patient. However, certain likely implications can be inferred from the known physiologic derangements. Given the elevated venous pressure and limited cardiac output in patients after Fontan, portal flow likely is diminished and portal vein saturation decreased, resulting in a dependency of the liver on HABR. This state likely exists even in patients with what are considered relatively favorable Fontan hemodynamics. As patients age and hemodynamic changes become more pronounced, it is very possible that the capability of HABR to compensate for diminished portal vein flow is exceeded, contributing to organ injury.

## Scope of the Problem: The Southampton Experience

The liver is the chief metabolic engine of the body. It effects xenobiotic and antimicrobial endotoxin clearance from the gut; coordinates carbohydrate, protein, lipid, vitamin, and hormone pathways; and importantly, plays a potentially key role in vasomotor dysregulation of the cardiovascular system when normal hepatic function is deranged.

**Fig. 1:**
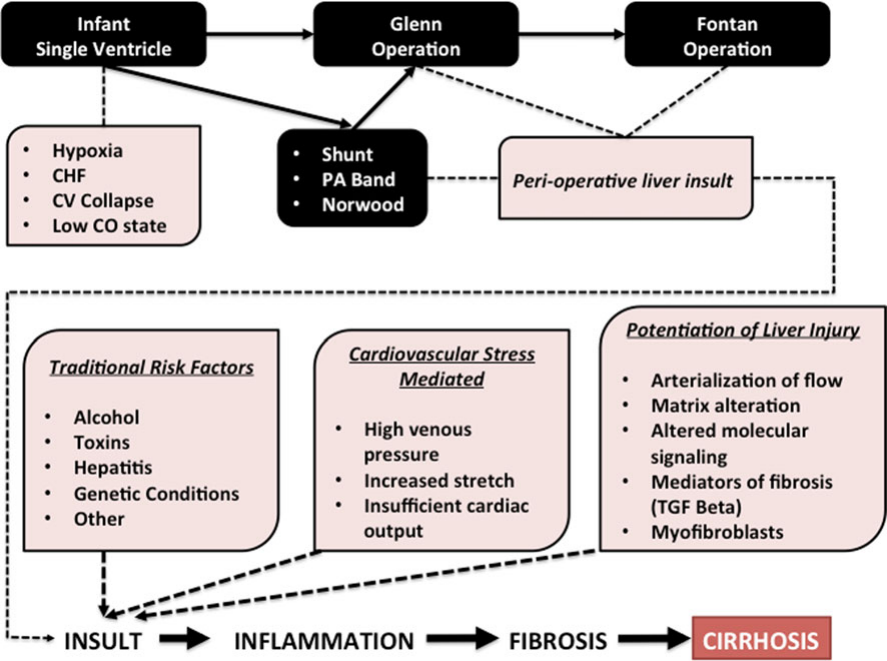
Potential course of the liver in a patient with the single-ventricle type of congenital heart disease

Potential discrete points of liver injury are commonplace in the natural and surgical history of patients undergoing single-ventricle palliative surgery. The initial clinical presentation may be marked by cardiovascular collapse, congestive heart failure, and marked hypoxemia in the infant with a single ventricle, factors well recognized to induce acute liver injury. Perioperative insults at the time of aortopulmonary shunts, Glenn shunts, or completion of the Fontan circulation also are well documented as causing ischemic liver insults. Chronic venous congestion characterizes the late Fontan circulation, and this combined with low cardiac output, particularly during periods of cardiovascular stress, is likely to induce hypoxic stress and acute chronic injury that may trigger inflammation, subsequent fibrosis, and potential cirrhosis (Fig. 1).

**Fig. 2:**
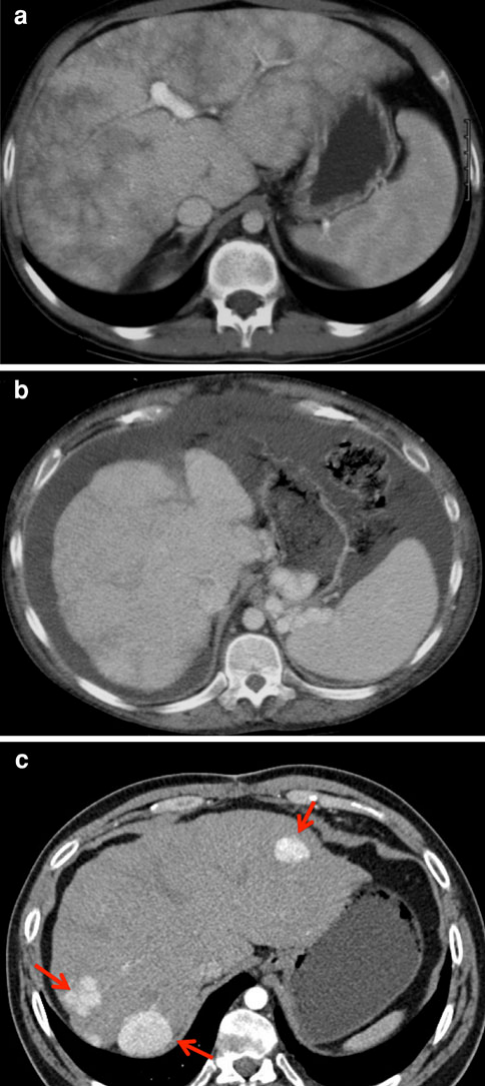
Contrast enhanced computed tomography (CT). Images a and b are venous-phase imaging. **a** Reticular pattern observed consistent histologically with broad scars. **b** Irregular nodular liver surface, perigastric varices, and splenomegaly. **c** Arterial phase demonstrating arterialized hypervascular nodules. Note the position in the liver periphery

The Southampton group and others have demonstrated gross alterations in liver structure, and more recently, in function, consistent with long-term hepatic insults [35]. Cross-sectional imaging using computed tomography (CT) or magnetic resonance imaging (MRI) for further characterization has demonstrated altered perfusion patterns that are zonal and reticular. The liver margin frequently is irregular, consistent with marked distortion of the liver’s gross architecture (Fig. 2a–c). Furthermore, investigators have observed positive markers of frank portal hypertension such as the presence of varices and splenomegaly.

Recently, the presence of arterialized nodules in the Fontan liver were described [6]. These occur with relatively high frequency. They typically reside in the outer margins of the liver and are seen in Fontan patients with higher venous pressures. These findings are consistent with changes seen in hepatic venous outlet obstruction such as Budd-Chiari syndrome. It is believed that they represent arterialization of the hepatic blood supply, an adverse adaption associated with portal venous deprivation of the liver parenchyma, perpetuating the underlying liver injury. Although these nodules are benign and pathologically identifiable as focal nodular hyperplasia, the initial characterization and follow-up treatment is challenging but vitally important because the main differential is hepatocellular carcinoma, which currently is increasingly reported.

Histologically, the adult Fontan liver phenotype is typified by massive and universal sinusoidal dilation [33]. This is associated with a diffuse felt-work of sinusoidal fibrosis, which is largely orcein negative, suggesting the potential for reversibility. Also frequently encountered, however, are broad fibrous spurs of orcein-positive scars, which in the more severe cases bridge the vessels, consistent with cirrhosis. These broad scars correspond macroscopically to the reticular pattern seen on CT. Quite disturbingly and of great concern, hepatocellular carcinoma in Fontan patients currently is documented clearly in the literature [25, 56].

The fundamental questions arising from these observations are how does such altered liver structure affect hepatic function, and what is the relevance in relation to clinical decision making? In this context, the Southampton group used indocyanine green clearance to document impaired liver function in Fontan patients, similar to a comparative group of compensated viral cirrhotic patients [28]. Both the disappearance and retention rates were significantly abnormal in Fontan patients. Contrary to what is observed in viral hepatitis–associated cirrhosis, the degree of dysfunction, however, was not proportional to the degree of fibrosis in the Fontan patients. Much more information is required to delineate this dysfunction further and its implications for the future prognosis and perioperative behavior of Fontan patients with demonstrated impaired function. In Southampton, it was decided on the basis of advanced liver disease not to operate on 3 of approximately 50 patients evaluated for Fontan revision surgery. One of these patients went on to experience hepatic encephalopathy and had hemodynamics ultimately and fatally dictated by decompensating cirrhosis. Velpula et al. [65] also documented acute variceal bleeding in the postoperative period of another patient who had undergone Fontan revision surgery.

It is safe to conclude that liver injury is universal in adult Fontan patients, whose liver is incorporated into the venous return to the lungs. The pattern of injury is very similar to other forms of cardiac cirrhosis in both its gross and histologic features. The important drivers appear to be high venous pressures, diminished cardiac output, and discrete insults pre- and perioperatively, as well as during late follow-up evaluation under circumstances of acute cardiovascular stress.

## Noninvasive Assessment of Liver Function and Fibrosis: Utility After the Fontan Operation

Noninvasive measures of hepatic function, structure, and fibrosis have been developed for clinical use and tailored to several different disease states. Testing can be focused on assessment of ongoing liver injury (test results that reflect liver damage or inflammatory states such as liver enzymes, FibroTest findings, and others) including measurements of true liver function (e.g., bilirubin, albumin, international normalized ratio [INR], indocyanine green clearance) and assessments of liver anatomy or fibrosis (e.g., routine imaging, elastography, diffusion-weighted MRI, contrast-enhanced ultrasound). Despite this plethora of available options, tests that distinguish accurately between milder gradations of liver disease in children are almost completely lacking [2], and this is especially true for the assessment of liver disease in the setting of congestive cardiac disease.

Evaluation of the degree of liver compromise in children and adults with congenital heart disease is of particular importance due to the significant impact of even mild liver disease on outcome from cardiac surgery, with overall mortality as high as 17 % [40] and an even higher mortality rate among those with more advanced liver disease.

It is clear that assessment of hepatic disease before cardiac surgery or transplantation is clinically important. Children with Fontan physiology pose particular difficulties for the clinician due to the nature of Fontan-associated liver disease (FALD) and the confounding features of Fontan physiology or treatment. The FALD condition is unique. Although variable, congestive hepatopathy appears to develop slowly in most of these children, often without obvious clinical features [3, 25]. Generally, elevation in liver enzymes is minimal or absent due to the lack of significant inflammation and cell death as fibrosis gradually accumulates.

Hepatic function is well preserved in FALD as fibrosis develops, as reflected by normal bilirubin. Serum albumin is normal in most children and if depressed, it is more commonly the result of protein-losing enteropathy rather than hepatic dysfunction. Clotting function, as reflected in the INR, also is normal unless confounded by warfarin therapy [3]. It is interesting to note that by the time of presentation to adult centers, those with failing Fontan physiology have a much higher rate of overt hepatic abnormalities, as shown by both standard blood testing and imaging [9, 36]. Screening tests that could identify earlier stages of hepatic fibrosis development would clearly be advantageous.

More sophisticated analyses of liver function such as the indocyanine green clearance test [17] or breath testing such as the 13C-caffeine [48] or 13C-aminopyrine [17] breath tests are intriguing possibilities that may be able to detect gradations of changes in liver function. Indocyanine green (ICG) is an inert dye eliminated exclusively by the liver through the bile without enterohepatic circulation. Clearance depends on hepatic extraction and bile flow and can be measured by serial blood testing or by transcutaneous colorometric monitoring similar to pulse oxymetry [67].

In a study of adults with chronic liver disease but no cardiac disease, ICG clearance and 13C-aminopyrine breath testing could distinguish between cirrhotic and noncirrhotic livers and accurately predict the risk of complications during the wait for a liver transplant. The 13C-caffeine breath test is based on the high oral bioavailability of caffeine as well as its nearly exclusive hepatic metabolism via demethylation by cytochrome P450 1A2 to carbon dioxide (CO_2_). Enrichment of breath 13-CO_2_ can thereby be used to calculate the hepatic metabolism of caffeine. This system, which is similar in concept to the aminopyrine breath test, has been studied in the setting of chronic hepatitis B virus (HBV) infection [47] and steato-hepatitis [48], and the results correlate with other parameters of hepatic function and fibrosis (at least in distinguishing cirrhotic from noncirrhotic disease). Alterations in caffeine and aminopyrine metabolism in cirrhosis may be hypothesized to occur due to altered first-pass metabolism rather than solely as a result of hepatocyte dysfunction. However, this may be a useful aspect for those with FALD.

Physical exam findings of portal hypertension such as caput medusa or splenomegaly often are lacking in patients with Fontan physiology, possibly due to altered hemodynamics, and platelet counts often are normal despite significant portal hypertension. When physical findings of portal hypertension do appear, consideration should be given to local anatomic causes such as portal vein thrombosis or stenosis after umbilical line placement in infants.

Radiologic assessment of liver fibrosis using a variety of methods also is available. Standard ultrasonography and CT scanning can assess nodularity with variable sensitivity. Doppler ultrasonography can be used to assess portal vein flow and the presence of collateral vessels as reflective of portal hypertension. Both nodularity from fibrosis and portal flow changes would be expected to be late findings and therefore not helpful in detecting signs of early hepatic compromise. Contrast-enhanced ultrasound, in which the transhepatic movement of microbubble contrast agents are traced with ultrasound, can improve detection of cirrhosis but again does not accurately distinguish earlier stages of fibrosis [39].

Transient elastography, an ultrasonographic method for surveying stiffness of a cylindrical hepatic sample, has advantages of rapidity, ease of use, and reliability among trained users. The use of elastography in Fontan-associated liver disease, however, is problematic in that any cause of altered hepatic stiffness (not only fibrosis) will have an impact on the results, particularly on vascular congestion [68], a universal finding in all Fontan subjects.

Friedrich et al. [21] studied 39 subjects with Fontan physiology using a variety of methods and found that 36 of the 39 subjects had abnormal stiffness by transient elastography, and 28 of 39 had fibrosis shown by the FibroTest (Paris, France). However, no liver biopsies were performed, and the issue of congestion as a complicating factor was not addressed. A comprehensive meta-analysis of 40 studies examining transient elastography in various clinical settings determined that though transient elastography theoretically has good sensitivity for cirrhosis, claims that it identifies various earlier stages of fibrosis have not been validated [62].

Diffusion-weighted MRI also has been proposed for evaluation of hepatic fibrosis but is plagued with similar problems. Sandrasegaran et al. [57] retrospectively reviewed the apparent diffusion coefficient (ADC) measured by MRI in subjects who also had undergone liver biopsy within 6 months after imaging. Although the ADC values for cirrhotic and noncirrhotic subjects differed significantly, the values did not differentiate among other fibrosis grades.

Current noninvasive tests are therefore relatively insensitive in any patient with liver disease, and the diagnostic dilemmas may be magnified in Fontan patients. It may be possible to develop a combination of complementary assessments that together more adequately predict the presence of liver disease and outcome of cardiac surgery for children with Fontan-associated liver disease. To do this, existing testing options must be considered carefully and prospective studies performed in children with Fontan physiology undergoing clinically indicated liver biopsy. Although the “gold standard” and best test available, liver biopsy itself has significant drawbacks ranging from the risk of the procedure (sedation in cardiac disease, bleeding) to the potential for sampling error and difficulty in interpretation of results. Therefore, the development of noninvasive alternatives would be a significant clinical advance.

## Liver Histology After the Fontan Operation and the Role of the Liver Biopsy

**Fig. 3:**
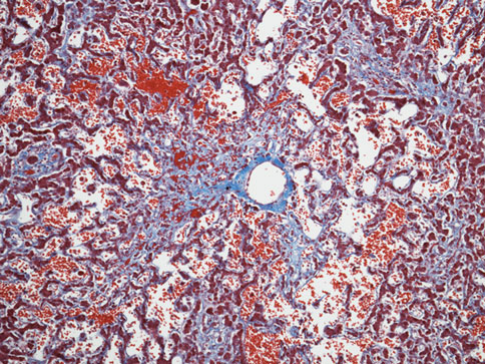
Liver specimen at autopsy from a 20-year-old man with long-standing right heart failure showing marked sinusoidal dilation and congestion. Bands of fibrosis extend from the central vein along the sinusoids. (Masson-Trichrome stain; magnification, ×100)

In the Fontan patient, elevated right-sided pressure is transmitted to the liver directly via the inferior vena cava and the hepatic veins and results in venous and perivenular sinusoidal congestion with atrophy of the liver plates in zone 3 (the centrilobular zone). With increasing right-sided failure, a combination of increasing right-sided pressure and low cardiac output results in hypoxemia, with further degeneration and eventual necrosis of zone 3 hepatocytes. The reticulin condenses, collagen formation increases, and perivenular fibrosis develops in the areas of necrosis, giving a venocentric pattern of fibrosis with bridging of adjacent hepatic veins (Fig. 3). As the disease advances, fibrous septae also link hepatic veins and portal tracts [7].

The histologic changes induced in the liver by the Fontan operation have been documented in a relatively small number of retrospective studies. Ghaferi and Hutchins [25] reported chronic passive congestion, centrilobular necrosis, and cardiac cirrhosis as single features or in combination at autopsy for nine patients who had undergone the Fontan procedure a few hours to 18 years previously. In their study, the severity of the hepatic changes observed correlated with the time elapsed after the Fontan operation and with right atrial pressures. The patients with the longest survival had cardiac cirrhosis, with the additional development of neoplastic transformation associated with the cirrhosis in two of the longest survivors.

In a report of 12 Fontan patients who underwent liver biopsies, Kiesewetter et al. [35] observed parenchymal atrophy, sinusoidal dilation, and fibrosis in all 12 patients, and cardiac cirrhosis in 7 of the patients. Broad scars were noted in eight of the patients. In their study, the severity of fibrosis, evaluated by “scoring” the biopsies, correlated with hepatic vein pressures and Fontan duration. These authors proposed a role for the liver biopsy in the management of patients with Fontan circulation.

In a follow-up publication from the same institution, the liver biopsies of 18 patients with Fontan circulation were evaluated using a semiquantitative scoring system, including a score for fibrosis developed for clinical studies of chronic hepatitis C [34]. The majority of the biopsy specimens had distorted architecture and fibrosis without any evidence of inflammation. The most marked feature of the biopsy specimens was sinusoidal dilation, which did not correlate with the degree of architectural distortion. The authors discussed the limitations of scoring fibrosis in this setting using a method originally developed for evaluation of another disease. In particular, pericellular sinusoidal fibrosis, observed in the majority of their biopsies, is not a feature evaluated in current scoring systems.

**Fig. 4:**
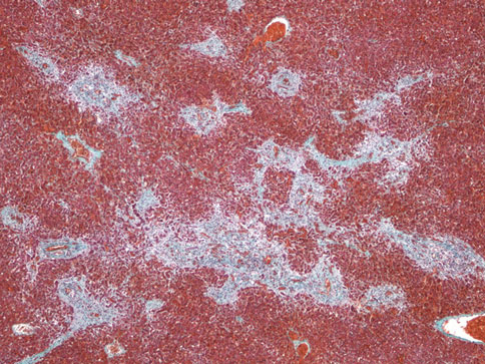
Liver at autopsy from a 2-year-old patient who died 1 day after a Fontan procedure. Extensive portal fibrosis is observed (Masson-Trichrome stain; magnification, ×40)

**Fig. 5:**
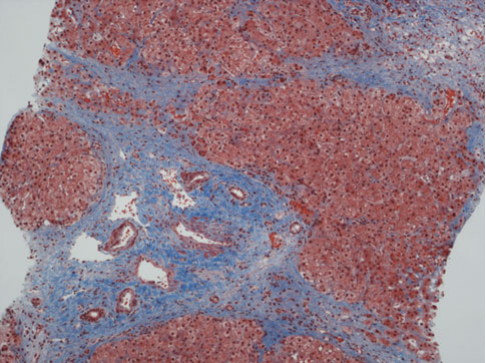
Liver biopsy from a 17-year-old girl 10 years after a Fontan procedure showing an expanded fibrotic portal tract with fibrosis in surrounding sinusoids. A broad scar is noted in the* upper right side* of the picture (Masson-Trichrome stain; magnification, ×100)

In a recent autopsy study from The Children’s Hospital of Philadelphia, portal-based fibrosis, not previously recognized in this population, was commonly observed and associated with time since the operation [58]. It also was seen in patients dying soon after the Fontan operation, suggesting that hepatic injury in this population may begin before the procedure (Figs. 4 and 5).

We can draw several conclusions from the foregoing discussion:

1. Liver fibrosis is a significant finding in patients with Fontan physiology.
2. The pathophysiology of the fibrosis may share similarities with typical cardiac hepatopathy, although the finding of significant portal-based fibrosis suggests involvement of additional factors.
3. The evaluation of liver fibrosis on biopsies may play an important role in the management of these patients, but staging fibrosis using current semiquantitative systems is problematic.

In current clinical practice, use of liver biopsy to grade the severity of inflammatory changes and to stage the extent of fibrosis plays an important role in the evaluation and management of patients with a wide range of diseases, including chronic hepatitis B and C, hemochromatosis, autoimmune hepatitis, and nonalcoholic fatty liver disease. Specimen size and quality must be adequate for interpretation to justify the inherent risk of the procedure. The biopsy must be large enough for an adequate number of portal tracts to be viewed, which is essential in the evaluation of liver architecture. An adequate number of portal tracts is proposed to be 10 or 11, the number being proportional to the biopsy size, which is recommended to be about 2 cm in length [52]. Short specimens may result in difficulties for patients with cirrhosis and could lead to failure in recognizing cirrhosis in up to 20 % of cases according to some studies [1, 51].

The width of the needle also is an important factor. In one study, both the grade and stage of viral hepatitis were significantly underestimated. An 18-gauge needle obtained samples 1 mm in diameter compared with wider specimens obtained using a 16-gauge needle [11].

Another important issue relates to the characterization of liver fibrosis in biopsies. Currently used methods of assessing liver damage and consequently, fibrosis, may not apply to the evaluation of liver biopsies in Fontan physiology.

First, scoring systems such as the Ishak, Scheuer, or METAVIR scoring systems were devised primarily to assess liver damage in chronic hepatitis, whereas no scoring system has been devised specifically for cardiac hepatopathy. These staging systems usually are part of a larger classification system that also includes assessment of inflammatory activity (grading). The liver damage in chronic hepatitis results primarily from a portal-based progressive inflammation-induced necrosis. These scoring systems reflect this portal-based pathology, whereas the damage in cardiac liver disease appears to be largely central and sinusoidal.

Second, these scoring systems rely primarily on architectural distortion and nodularity and do not relate specifically to the amount of fibrosis in the liver sample. Given that fibrosis in Fontan livers may be both central and portal based, an assessment of the overall amount of fibrosis may be more relevant.

Third, these scoring systems are descriptive categories and not a set of numbers arithmetically related to each other. That is, stage 2 is not half of stage 4.

Finally, the histopathologic assessment of fibrosis in liver samples usually is performed with trichrome or reticulin stains, which do not correspond quantitatively to the amount of collagen in the biopsy. Histochemistry using Sirius red, which has an affinity for collagen types 1 and 3, may be more suitable for quantitative assessment [23]. Evaluation of fibrosis using Sirius red staining in combination with digital image analysis may offer a quantitative assessment of fibrosis that does not depend on an architectural-based semiquantitative scoring system.

Image analysis has been used in a few studies evaluating changes in hepatic fibrosis after alpha-interferon therapy. A recent study using computerized quantification of liver fibrosis in biliary atresia found that the volume of fibrosis correlated with transplant-free survival, whereas the Ishak scoring system showed no correlation [46]. In another study correlating fibrosis with hepatic venous pressure in patients with hepatitis C, the amount of collagen assessed by digital image analysis was a better histologic correlate with hepatic venous pressure than the Ishak stage [8].

## Mechanisms of Hepatic Fibrosis in Patients With the Fontan Circulation: The Questions

Hepatic fibrosis refers to the presence of excess and abnormal extracellular matrix (ECM)—scar tissue—in the liver. Although significant breakthroughs in understanding the general mechanisms of fibrosis have occurred over the last two decades [18], the rodent model systems and human diseases typically studied differ significantly from Fontan-associated liver disease, which has several unusual features. As demonstrated in a published study of histologic findings in livers of patients with a Fontan circulation, inflammation and hepatocyte damage, seen in most other forms of fibrosis and important contributors to fibrosis progression, are absent. Additionally, as noted earlier, although many forms of fibrosis are located in the parenchyma or biliary regions, fibrosis in Fontan patients was found to be pericentral and sinusoidal.

The following sections discuss four areas relating to fibrosis in Fontan patients that would benefit from further research specific to this condition.

### The Nature of the ECM

Fibrosis is notable for changes in the amount, distribution, and quality of ECM. These changes vary according to the etiology of fibrosis and potentially determine the nature of fibrosis progression as well as the response to treatment.

The newly deposited matrix in a fibrotic liver comprises a variety of matrix proteins. Of these, the best studied are the fibrillar collagens (particularly types 1 and 3), which are the major components of the fibrotic scar. In addition to increases in expression, these collagens undergo significant cross-linking as fibrosis progresses. This cross-linking, which is mediated by enzymes in the lysyl oxidase and tissue transglutaminase families [32, 50], enhances the mechanical stiffness of the collagens and may regulate their susceptibility to degradation [15, 64]. Understanding the nature and regulation of cross-linking in fibrosis may be important to identifying specific antifibrotic therapies and determining the potential for fibrosis to regress.

Fibronectin, a normally abundant ECM protein, increases further in fibrosis, particularly the cellular fibronectin isoform and many of its multiple splice variants. Although the functions of these splice variants are debated, they may include facilitation of angiogenesis. Other matrix proteins upregulated in fibrosis include a variety of proteoglycans, most notably members of the small leucine-rich family of proteoglycans that play a role in collagen organization and elastin, which undergoes cross-linking similar to collagen. Fibrotic areas of Fontan livers stain with the dye orcein, which binds to elastin and to cross-linked collagen, suggesting maturity of the scar and chronicity of the injury [31, 34].

There are multiple matrix-related questions that merit further examination in the livers of patients with a Fontan circulation: What is the distribution of the excess abnormal ECM in Fontan livers (pericentral, sinusoidal, periportal), and does this change as fibrosis progresses? How much elastin is present, and what is its distribution? Does this reflect maturity of the scar, or is it a function of the localization of the disease process? How highly cross-linked are collagens and elastin in Fontan livers, and which enzymes mediate their cross-linking? Do other matrix proteins highly expressed in Fontan livers exist that are relevant to collagen mechanics or angiogenesis?

### Myofibroblasts: The Effector Cells of Fibrosis

Myofibroblasts, defined operationally as cells that express α-smooth muscle actin de novo, are the matrix-producing cells of the fibrotic liver. They are contractile, motile, and proliferative cells responsible for the majority of abnormal ECM deposition in tissue fibrosis. Myofibroblasts differentiate from a variety of precursor cells. In the liver, the best described are hepatic stellate cells, located in the space of Disse adjacent to the sinusoids, and portal fibroblasts, located in the periportal space. Hepatic stellate cells play multiple roles in the liver. In additional to being mediators of fibrosis, they also function as pericytes in angiogenesis and are highly contractile cells that potentially regulate sinusoidal hemodynamics [19]. Portal fibroblasts are the “first responders” after biliary injury and thus play a critical role in biliary fibrosis [13, 37]. It is possible that fibroblasts around the central vein play a similar role in pericentral fibrosis (e.g., in the Fontan liver) [29]. However, these cells have not been well studied.

Myofibroblasts derived from hepatic stellate cells and portal fibroblasts can be differentiated on the basis of marker protein expression [5]. They also appear to differ functionally in their response to various stimuli and their profiles of ECM expression [29], with portal fibroblasts, for example, expressing elastin [38]. Much of the known information about the characterization and function of liver myofibroblasts is derived from the study of isolated primary cells in culture. Both hepatic stellate cells and portal fibroblasts activate to myofibroblasts in culture, and the study of enhancers and inhibitors of this process has proved to be invaluable in understanding the dynamics of myofibroblasts in fibrosis. Importantly, however, although the isolation of relatively pure populations of portal fibroblasts by our group and others has been described [38], this population may include pericentral fibroblasts that cannot be differentiated from portal fibroblasts.

The myofibroblast populations involved in Fontan-associated fibrosis are not known. In particular, although hepatic stellate cells likely play an important role in sinusoidal fibrosis, the myofibroblasts responsible for pericentral fibrosis have not been well defined. Relevant questions about the myofibroblast population in Fontan livers include the following: Which cells are the main myofibroblast precursor cells? Are pericentral fibroblasts involved? Does this change as fibrosis progresses? What are pericentral fibroblasts? Are they functionally similar to portal fibroblasts, and do they deposit elastin? Are hepatic stellate cells involved in the sinusoidal fibrosis of Fontan livers? Does this have implications for sinusoidal hemodynamics or angiogenesis?

### Factors Causing Activation of Precursor Cells to Myofibroblasts

Multiple factors have been shown to mediate myofibroblast activation, most of which have been identified through in vitro studies. These include the soluble transforming growth factor-β (TGFβ), which appears to be a central mediator in all forms of organ fibrosis and is downstream of many other soluble factors found to influence the process of activation [27].

Mechanical tension also is required for activation of myofibroblasts [60]. Tension can be generated through matrix stiffness, which regulates the differentiation of both hepatic stellate cells and portal fibroblasts to myofibroblasts [38, 44]. In the fibrotic liver, increased deposition of rigid ECM proteins such as the fibrillar collagens influences the local mechanical environment. Increases in collagen cross-linking due to lysyl oxidase may enhance these changes [22]. In some contexts, the release of active TGFβ from its latent form requires a stiff environment, suggesting that matrix, the mechanical environment, and TGFβ are interrelated [66].

The role of mechanical forces other than matrix stiffness in myofibroblast differentiation is not well understood. In particular, whether the elevated outflow tract and sinusoidal pressure that characterize many Fontan livers can facilitate the activation of myofibroblast precursor cells has not been well studied. One publication reported that culturing hepatic stellate cells under conditions of elevated hydrostatic pressure resulted in enhanced hepatic stellate cell upregulated α-smooth muscle actin expression and collagen deposition [43]. Also unknown is whether such mechanical forces can influence TGFβ activation. Increased pressure in the biliary tree as a result of bile duct ligation leads to fibrosis, suggesting that such mechanical forces can influence myofibroblast behavior [30].

Hypoxia has recently been shown to regulate hepatic stellate cell function, both directly and indirectly, and may be at the crossroads between fibrosis and angiogenesis [61]. The relative portal venous hypoxia in some Fontan patients may provide an additional mechanism for liver fibrosis.

Thus, understanding why fibrosis develops in patients with a Fontan circulation requires answering several key questions: What is the role of increased central vein and sinusoidal pressure in myofibroblast activation? Does it function similarly to increased matrix stiffness? Is increased pressure in the central vein transmitted to the sinusoids, and does it have effects on peri-sinusoidal myofibroblast precursors (hepatic stellate cells)? Do Fontan-associated mechanical changes have an effect on the release of active TGFβ from its latent form? What are the implications of improved venous outflow pressure for fibrosis in Fontan livers? Does hypoxia contribute to any of the changes seen in Fontan livers?

### The Regression of Fibrosis and, Potentially, Cirrhosis

Regression as opposed to resolution of fibrosis unquestionably occurs when the underlying chronic insult is removed [16, 20]. This has been demonstrated in multiple human diseases and in animal models and is thought to require apoptosis of myofibroblasts as well as degradation and remodeling of the extracellular matrix. Whether regression of cirrhosis occurs is debatable. Although early cirrhosis likely can regress, the heavily cross-linked collagen and elastin of late cirrhosis may be resistant to remodeling [31]. Additionally, late cirrhosis is characterized by significant vascular changes, including marked shunting, which are responsible for many of the clinical manifestations of the disease. These may persist even in the setting of architectural remodeling [49]. Thus, there may be a “point of no return” along the spectrum of cirrhosis. Where that point is and whether it varies according to the etiology of the disease are not known.

Regression is a particularly relevant issue for clinicians caring for Fontan patients who may face the issue of heart versus heart/liver transplantation. Other speakers and discussants at the symposium suggested that Fontan patients with liver disease, even without cirrhosis, are at high risk for liver decompensation in the setting of a heart transplant. Aside from these acute issues, however, it is possible that patients with fibrosis and early cirrhosis whose circulation is normalized with a heart transplant could undergo clinically relevant regression of liver disease, avoiding the need for a liver transplant.

Key questions underlying regression in the Fontan-affected liver include the following: What are the vascular changes in Fontan-associated liver disease? When do they occur and do they reverse in the setting of matrix remodeling? At what point in the progression of Fontan fibrosis does regression become unlikely? Is this related to the qualities of the deposited matrix (elastin content, degree of cross-linking)? If the factors (potentially mechanical) that induce fibrosis in patients with the Fontan circulation were reversed, would liver disease regress? What implications does this have for the treatment of circulatory abnormalities in patients with early liver disease? What implications does it have for fibrotic and cirrhotic patients undergoing heart transplantation?

## Where Do We Go from Here? A Recommendation for Systematic Evaluation of the Liver After the Fontan Operation

In our view, an abundance of data confirms the presence of an indolent but devastating pathologic process affecting the liver after the Fontan operation. Although invasive and still not ideal, liver biopsy appears to be the most accurate means for assessing the degree of hepatic pathology present. Realizing the potential insult and ongoing damage, our group consensus after the Symposium is to recommend initiation of a program for evaluation of liver status through tissue biopsy analysis, framed within the context of a contemporary detailed characterization of cardiovascular status. We plan to evaluate all patients approximately 10 years after the Fontan operation. The evaluation will include a liver biopsy combined with cardiovascular assessment through cardiac catheterization and cardiac magnetic resonance imaging.

Although many questions remain, it is clear that liver damage is taking place in survivors of Fontan surgery. Is a program of proactive liver biopsy and cardiac evaluation after Fontan operation to be considered investigational research? In our view, assessing the degree of liver pathology for any individual patient may be considered a new clinical care approach and is not research.

Individual patient care will be dictated based on the findings. For example, if significant bridging fibrosis is discovered within the context of elevated central venous pressure, then initiation of pulmonary vasodilator therapy may be considered. Such patients would be “flagged” and referred to the care of hepatology specialists for closer serial follow-up evaluation and management. Furthermore, patients with extensive liver pathology could be considered for possible heart transplantation therapy or could be at the top of the list for consideration of more innovative therapies such as mechanical assist and support devices currently in development.

No doubt there will be interest in creating generalizable knowledge as the clinical experience with detailed liver evaluation in our Fontan population grows. In particular, development of noninvasive correlates with the liver biopsy findings is essential. Creation of a detailed registry of patients who will undergo hepatic and cardiovascular evaluation at this decade milestone will be of great importance. We endorse a shift in philosophy, from one of reactivity to the onset of clinical complications to a proactive, systematic clinical investigation of end-organ function in our survivors of the Fontan operation. Liver pathology is present, likely progressive, and demands a better understanding both for the individual patient and for the community of survivors, which is rapidly expanding.
